# The association between sodium glucose cotransporter‐2 inhibitors vs dipeptidyl peptidase‐4 inhibitors and renal outcomes in people discharged from hospital with type 2 diabetes: A population‐based cohort study

**DOI:** 10.1111/1753-0407.13507

**Published:** 2024-04-10

**Authors:** Kate E. D. Ziser, Stephen Wood, George S. Q. Tan, Jedidiah I. Morton, Jonathan E. Shaw, J. Simon Bell, Jenni Ilomaki

**Affiliations:** ^1^ Centre for Medicine Use and Safety, Faculty of Pharmacy and Pharmaceutical Sciences Monash University Melbourne Victoria Australia; ^2^ Clinical Diabetes and Epidemiology, Baker Heart and Diabetes Institute Melbourne Victoria Australia

**Keywords:** acute kidney injury, chronic, cohort studies, kidney failure, sodium‐glucose transporter 2 inhibitors

## Abstract

**Background:**

We investigated the association between post‐hospital discharge use of sodium glucose cotransporter‐2 inhibitors (SGLT‐2is) compared to dipeptidyl peptidase‐4 inhibitors (DPP‐4is) and the incidence of hospitalization for acute renal failure (ARF) and chronic kidney disease (CKD) in people with type 2 diabetes.

**Methods:**

We conducted a retrospective cohort study using linked hospital and prescription data. Our cohort included people aged ≥30 years with type 2 diabetes discharged from a hospital in Victoria, Australia, from December 2013 to June 2018. We compared new users of SGLT‐2is with new users of DPP‐4is following discharge. People were followed from first dispensing of a SGLT‐2i or DPP‐4i to a subsequent hospital admission for ARF or CKD. We used competing risk models with inverse probability of treatment weighting (IPTW) to estimate subhazard ratios.

**Results:**

In total, 9620 people initiated SGLT‐2is and 9962 initiated DPP‐4is. The incidence rate of ARF was 12.3 per 1000 person‐years (median years of follow‐up [interquartile range [IQR] 1.4 [0.7–2.2]) among SGLT‐2i initiators and 18.9 per 1000 person‐years (median years of follow‐up [IQR] 1.7 [0.8–2.6]) among DPP‐4i initiators (adjusted subhazard ratio with IPTW 0.78; 95% confidence interval [CI] 0.70–0.86). The incidence rate of CKD was 6.0 per 1000 person‐years (median years of follow‐up [IQR] 1.4 [0.7–2.2]) among SGLT‐2i initiators and 8.9 per 1000 person‐years (median years of follow‐up [IQR] 1.7 [0.8–2.6]) among DPP‐4i initiators (adjusted subhazard ratio with IPTW 0.83; 95% CI 0.73–0.94).

**Conclusions:**

Real‐world data support using SGLT‐2is over DPP‐4is for preventing acute and chronic renal events in people with type 2 diabetes.

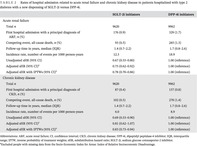

## Graphical abstract

Highlights

- What are the new findings?

This was the first Australian study using real‐world data to show SGLT‐2is reduce ARF and CKD compared to DPP‐4is in people with type 2 diabetes. The rates of hospital admissions for ARF and CKD in people with type 2 diabetes were 22% and 17% lower, respectively, among SGLT‐2i initiators compared to DPP‐4i initiators.

- How might this have an impact on clinical practice in the foreseeable future?

Our study suggests that benefits of SGLT‐2is apparent in clinical trials, in preventing acute and chronic renal events in people with type 2 diabetes, are reflected in real‐world clinical practice.

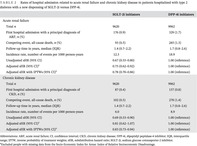

## 1 INTRODUCTION

Initiation of sodium‐glucose cotransporter 2 inhibitors (SGLT‐2is) has been linked to an initial decline in estimated glomerular filtration rate (eGFR).^1^, ^2^, ^3^, ^4^, ^5^, ^6^ However, SGLT‐2is have also been associated with reduced risk of acute renal failure (ARF) in clinical trials^1^, ^2^, ^3^, ^7^, ^8^ and have been shown to slow decline in eGFR in people with type 2 diabetes following an initial (and reversible) decline.^4^, ^9^, ^10^, ^11^, ^12^ Several large clinical trials have shown SGLT‐2is slow progression of chronic kidney disease (CKD).^9^, ^10^, ^12^, ^13^, ^14^

Replicating findings observed in clinical trials in a real‐world population is important, as clinical trial samples are seldom representative of the broader population with diabetes.^15^, ^16^, ^17^ Indeed, most estimates of the effect of SGLT‐2is on ARF are drawn from high‐risk populations, and their generalizability to the broader population with type 2 diabetes is unclear. For example, the Dapagliflozin and Prevention of Adverse Outcomes in Chronic Kidney Disease (DAPA‐CKD) trial included individuals with demonstrated baseline CKD, a median age of 62, and no participants over the age of 74, with no stratification of older people with respect to incidence of adverse renal events.^10^

Other observational studies have reported on whether SGLT‐2is are associated with reduced risk of ARF.^5^, ^7^, ^18^, ^19^, ^20^, ^21^ There is a lack of consensus and limitations among these studies as some report no association and others show a reduction in ARF with SGLT‐2i initiation.^5^, ^7^, ^18^, ^19^, ^20^, ^21^ Some previous studies that reported no significant effect of SGLT‐2is on ARF have used any oral glucose lowering drug,^19^ glucagon‐like peptide 1 (GLP1) agonist,^20^ DPP‐4i/GLP1 agonist/gliclazide,^4^ or no use as a comparator group.^22^ Other observational studies have reported 21%,^5^ 21%,^18^ and 53%^5^, ^18^ reductions in rates of ARF with SGLT‐2i initiation versus dipeptidyl peptidase‐4 inhibitor (DPP‐4i) initiation.

The objective of this study was to investigate the association between post‐hospital discharge initiation of SGLT‐2i compared to DPP‐4i and the incidence of subsequent hospital admission for ARF and CKD in people with type 2 diabetes.

## 2 METHODS

### 2.1 Data sources

We conducted a retrospective cohort study using linked hospital and prescription data from the state of Victoria, Australia. Victoria is the second most populous state in Australia with a population of 6.68 million. We included all people with type 2 diabetes discharged from a public or private hospital in Victoria from January 12, 2013 to June 30, 2018. We used data from the Victorian Admitted Episodes Dataset (VAED), which contains data on administrative and demographic information, as well as medical diagnoses and procedures during hospital admission. The VAED was linked to data from the Pharmaceutical Benefits Scheme (PBS) for information on medication dispensing and the National Death Index (NDI) for dates of death. The PBS is an Australian government scheme that provides medicines at a subsidized price for Australian citizens and people from countries with reciprocal agreements. Most medications dispensed from community pharmacies or upon discharge from hospital in Australia are subsidized under the PBS, this figure being 93.5% in the 2019–2020 financial year.^23^, ^24^ The NDI contains dates and causes of death for deaths occurring within Australia.

This study was approved by the Australian Institute of Health and Welfare Ethics Committee (approved amendment to EO2018/4/468) and Monash University Human Research Ethics Committee (Project ID 14339).

### 2.2 Study population and exposures

The study cohort was defined as people aged ≥30 years and discharged from a Victorian public or private hospital with an *International Classification of Diseases, Tenth Revision, Australian Modification* (ICD‐10‐AM) code indicative of type 2 diabetes (E11) between January 12, 2013 and June 30, 2018.

**FIGURE 1:**
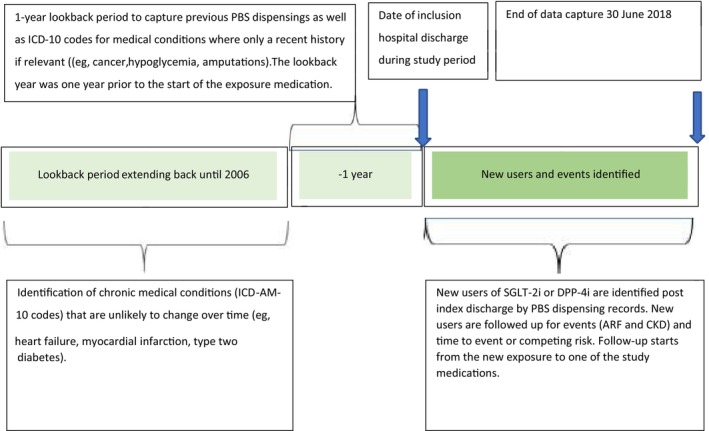
Illustration depicting the study design. ARF, acute renal failure; CKD, chronic kidney disease; DPP‐4i, dipeptidyl peptidase‐4 inhibitor; ICD‐10‐AM, *International Classification of Diseases, Tenth Revision, Australian Modification*; PBS, Pharmaceutical Benefits Scheme; SGLT‐2i, sodium glucose cotransporter‐2 inhibitor.

The index admission was defined as the first hospital admission during the study period among individuals with a diagnosis code for type 2 diabetes (see Table A1 for cause of index hospitalization). The index date was defined as the date when either an SGLT‐2i or DPP‐4i was initiated on or after the discharge date (Figure 1). Users who were dispensed the DPP‐4i linagliptin were excluded as this is the preferred DPP‐4i in reduced renal function. PBS data were available from 2006. This allowed the date of first supply to be ascertained because neither SGLT‐2i nor DPP‐4i were available in Australia prior to 2006. Anatomical Therapeutic Chemical codes were used to identify first SGLT‐2i and DPP‐4i dispensings (see Table A2). Only new users of SGLT‐2i or DPP‐4i were considered. This meant that people were included in the study only if they had no dispensing record of SGLT‐2i and DPP‐4i prior to the index date. People receiving both an SGLT‐2i and a DPP‐4i for the first time on the same day were excluded. DPP‐4is were chosen as the comparator to reduce confounding by disease severity as both SGLT‐2i and DPP‐4i are second‐line agents for type 2 diabetes in Australia.^25^

**FIGURE 2:**
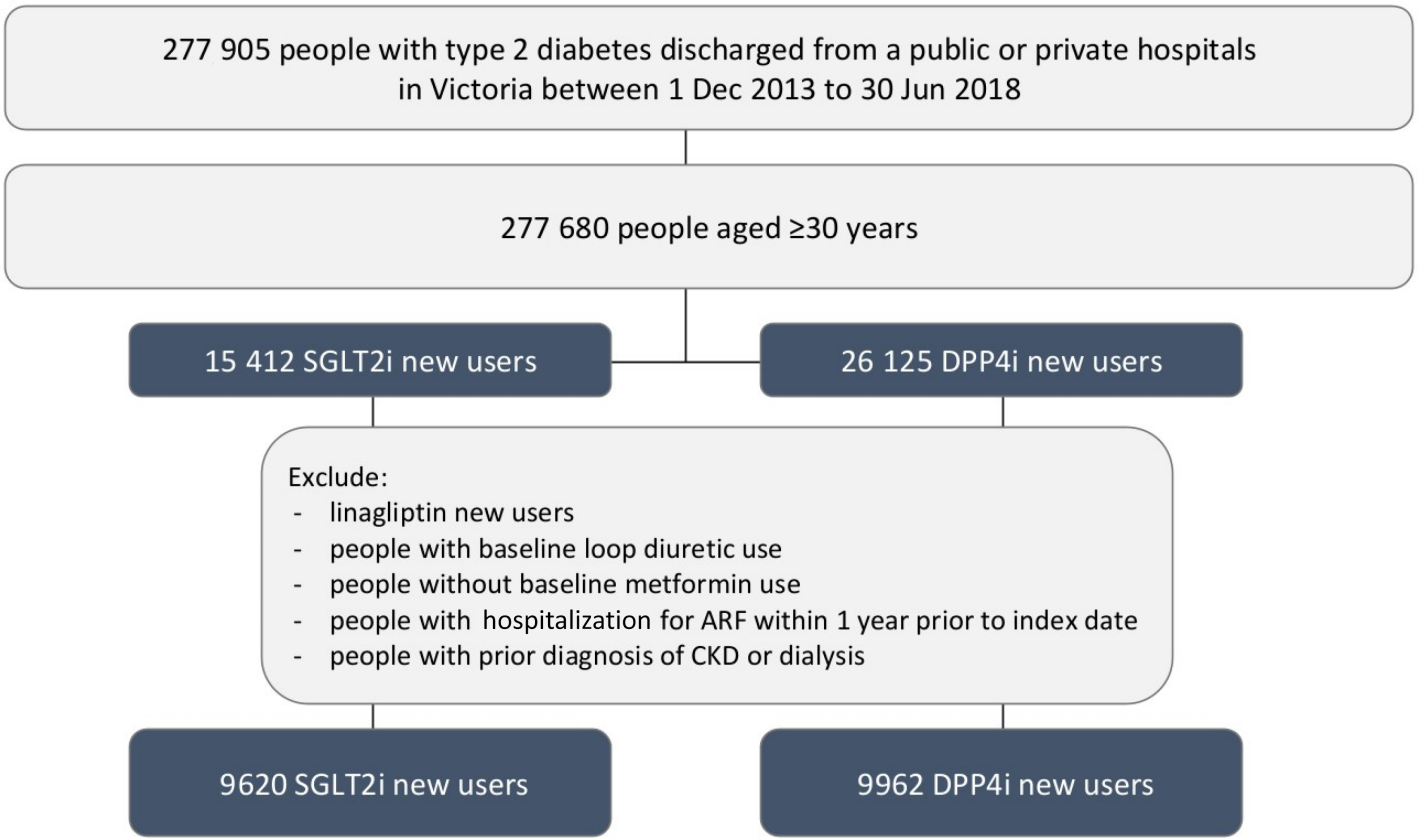
Flow chart for selection of patients. ARF, acute renal failure; CKD, chronic kidney disease; DPP‐4i, dipeptidyl peptidase‐4 inhibitor; SGLT‐2i, sodium glucose cotransporter‐2 inhibitor.

The cohort was restricted to people using metformin within 1 year prior to the index date and excluded people with use of loop diuretics within 1 year prior to the index date, with hospitalization for ARF within 1 year prior to index date, or a prior diagnosis of CKD or dialysis since 2006 to index date. Our rationale for this was to minimize channeling bias as at the time of the study SGLT‐2i were contraindicated in severe renal impairment. Figure 2 shows the flow chart for selection of patients.

### 2.3 Outcomes

Outcomes included first hospital admission with a primary diagnosis code for ARF (ICD‐10‐AM code N17) or CKD (ICD‐10‐AM code N18). People were followed up from the index date until the first outcome, death, or the end of data period (June 30, 2018).

### 2.4 Covariates

As covariates we considered baseline medication use, comorbidities, socioeconomic disadvantage, type 2 diabetes severity, and year of initiation of exposure medication. Baseline medication use was identified using PBS dispensing records within 1 year prior to the index date (inclusive) (Table A2). Comorbidities were identified from the VAED using ICD‐10‐AM codes from hospitalizations from 2006 until the index date (Table A3).^26^ Socioeconomic disadvantage was measured using the Socio‐Economic Indexes for Areas: Index of Relative Socioeconomic Disadvantage (IRSD).^27^ An IRSD quintile score of 1 to 5 was generated for each patient based on their postcode when they had the initial exposure medication commenced, the lower the score, the higher level of relative socioeconomic disadvantage.^28^ A modified version of the Diabetes Complication Severity Index (DCSI) was used to assess type 2 diabetes severity. The DCSI uses ICD‐10 codes from hospital admissions data over a 1‐year lookback period from the index date to quantify the effect of diabetes on multiple organ systems.^12^ The higher the score, the more severe the type 2 diabetes complications.^25^

### 2.5 Statistical analysis

Baseline characteristics of SGLT‐2i and DPP‐4i initiators are presented as frequencies and percentages or median and interquartile range (IQR). We also present the number and proportion of people with the outcomes and median time (with IQR) of follow‐up. Competing risk analyses with inverse probability of treatment weights (IPTWs) were conducted to estimate the risk of hospital admission with ARF or CKD using separate models. IPTWs were used to balance the baseline characteristics of the exposure and comparator groups thus minimizing the effects of prescriber bias.^29^ The propensity score estimation model included age, sex, calendar year, other medication use (refer to Table A2 for a complete list), comorbidities (refer to Table A3 for a complete list), socioeconomic disadvantage, and diabetes severity. All variables presented in Tables A2 and A3 were included in the models. We conducted competing risk analyses using weighted Fine and Gray's subdistribution hazard models to estimate the subhazard ratios (sHRs) and 95% confidence intervals (CI) for ARF and CKD. All‐cause death was considered as the competing event and censoring occurred at the end of the study period for those with event‐free survival. We conducted all analyses using SAS version 9.4 (SAS Institute Inc., Cary, NC, USA).

## 3 RESULTS

### 3.1 Patient characteristics

In total, there were 9620 new users of SGLT‐2is and 9962 new users of DPP‐4is who met the inclusion criteria of baseline use of metformin and nil history of CKD or ARF (out of 15 412 SGLT‐2i initiators and 26 125 DPP‐4i initiators). The proportion of females was 41.4% in the SGLT‐2i group and 42.2% in the DPP‐4i group. A higher proportion of those dispensed SGLT‐2is compared to DPP‐4is were aged 30–59 years (Table 1). Overall, 2.2% of people dispensed SGLT‐2is and 6.7% of people dispensed DPP‐4is were 80 years or older. The proportions of SGLT‐2i and DPP‐4i initiations over time were similar, with the number of initiations increasing over time in both groups. A small number of initiators (1.1% in the SGLT‐2i group and 0.9% in the DPP‐4i group) had an index discharge in 2018. Aldosterone antagonist use was lower in new users of SGLT‐2is compared to DPP‐4is (2.0% and 5.6% respectively). Prevalence of comorbidities were similar in new users of SGLT‐2i compared to DPP‐4i. Median time (IQR) from hospital discharge until start of treatment was slightly longer in new users of SGLT‐2i (1.5 years [0.8–2.4]) compared to DPP‐4i (1.3 years [0.6–2.2]) (Table 2).

**TABLE 1:** Baseline characteristics of patients hospitalized with type 2 diabetes with a new dispensing of SGLT‐2i or DPP‐4i on or after their index admission date.

Total	SGLT‐2i initiators (*n* = 9620)	DPP‐4i initiators (*n* = 9962)
Baseline characteristics		
Age, years, *n* (%)		
30–59	4276 (44.5)	3786 (38.0)
60–69	3603 (37.5)	3296 (33.1)
70–79	1531 (15.9)	2215 (22.2)
80+	210 (2.2)	665 (6.7)
Sex, *n* (%)		
Female	3987 (41.4)	4206 (42.2)
Year of index discharge, *n* (%)		
2013	395 (4.1)	554 (5.6)
2014	4017 (41.8)	4679 (47.0)
2015	2736 (28.4)	2621 (26.3)
2016	1639 (17.0)	1447 (14.5)
2017	727 (7.6)	574 (5.8)
2018 (6 months of data available)	106 (1.1)	87 (0.9)
Year of SGLT‐2i or DPP‐4i initiation, *n* (%)		
2013/2014^a^	178 (1.8)	948 (9.5)
2015	1677 (17.4)	1953 (19.6)
2016	2737 (28.5)	2844 (28.6)
2017	3451 (35.9)	2799 (28.1)
2018 (6 months of data available)	1577 (16.4)	1418 (14.2)
Median time from hospital discharge date until start of treatment in years, median (IQR)	1.5 (0.8–2.4)	1.3 (0.6–2.2)
Diabetes complications severity index^b^, *n* (%)		
0	8821 (91.7)	9113 (91.5)
1	558 (5.8)	543 (5.5)
≥2	241 (2.5)	306 (3.1)
Medication use up to 1 year prior to SGLT‐2i or DPP‐4i initiation, *n* (%)		
ACE inhibitors/ARB	4309 (44.8)	4360 (48.6)
Beta‐blockers	1477 (15.4)	1516 (24.5)
Calcium channel blockers	933 (9.7)	957 (15.3)
Lipid‐lowering medications	4557 (47.4)	4735 (52.3)
Aldosterone antagonists	191 (2.0)	131 (5.6)
Digoxin	80 (0.8)	133 (3.7)
Oral anticoagulant	120 (1.3)	172 (1.7)
Antiplatelet	1007 (10.5)	1052 (10.6)
Antipsychotics	284 (3.0)	333 (3.3)
Anticholinergic	14 (0.2)	42 (0.4)
Medical conditions prior to index date, *n* (%)		
Unstable angina	506 (5.3)	400 (4.0)
Angina pectoris	540 (5.6)	451 (4.5)
Peripheral vascular disease	248 (2.6)	276 (2.8)
Myocardial infarction	682 (7.1)	573 (5.8)
Heart failure	152 (1.6)	162 (1.6)
Hypertension	2889 (30.0)	2630 (26.4)
Atrial fibrillation	509 (5.3)	582 (5.8)
Stroke	267 (2.8)	347 (3.5)
Chronic obstructive pulmonary disease	342 (3.6)	373 (3.7)
Cancer^c^	322 (3.4)	479 (4.8)
Severe hypoglycemia	15 (0.2)	19 (0.2)
Diabetes polyneuropathy	639 (6.6)	526 (5.3)
Diabetic eye disease	1591 (16.5)	1633 (16.4)
Diabetic foot	429 (4.5)	345 (3.5)
Other diabetic complications	4835 (50.3)	4066 (40.8)
Amputation	30 (0.3)	24 (0.2)
Acidosis^c^	32 (0.3)	34 (0.3)
IRSD quintile^d^, *n* (%)		
1 (highest level of relative socioeconomic disadvantage)	2249 (23.4)	2338 (23.5)
2	1841 (19.2)	1991 (20.0)
3	2142 (22.3)	2209 (22.2)
4	2006 (20.9)	1999 (20.1)
5 (lowest level of relative socioeconomic disadvantage)	1369 (14.3)	1415 (14.2)
Missing	13 (<0.1)	10 (<0.1)

Abbreviations: ACEI, angiotensin converting enzyme inhibitor; ARB, angiotensin 2 receptor blocker; DPP‐4i, dipeptidyl peptidase‐4 inhibitor; IQR, interquartile range; SGLT‐2i, sodium glucose cotransporter‐2 inhibitor.

^a^ The 2 years were combined to avoid cells with small numbers (less than 6) as per the Australian Institute of Health and Welfare requirements.

^b^ A modified version of the Diabetes Complications Severity Index (DCSI) was used to assess diabetes severity.^23^

^c^ Medical conditions from a 1‐year lookback (all other medical conditions were taken from the total lookback since 2006).

^d^ Socioeconomic disadvantage was measured using the Socio‐Economic Index for Areas: Index of Relative Socioeconomic Disadvantage (IRSD).^27^

**TABLE 2:** Rates of hospital admission related to acute renal failure and chronic kidney disease in patients hospitalized with type 2 diabetes with a new dispensing of SGLT‐2i versus DPP‐4i.

	SGLT‐2i initiators	DPP‐4i initiators
Acute renal failure		
Total *n*	9620	9962
First hospital admission with a principal diagnosis of ARF, *n* (%)	176 (0.9)	329 (1.7)
Competing event, all‐cause death, *n* (%)	93 (0.5)	245 (1.3)
Follow‐up time in years, median (IQR)	1.4 (0.7–2.2)	1.7 (0.8–2.6)
Incidence rate, number of events per 1000 person‐years	12.3	18.9
Unadjusted sHR (95% CI)	0.67 (0.55–0.80)	1.00 (reference)
Adjusted sHR (95% CI)^a^	0.75 (0.62–0.92)	1.00 (reference)
Adjusted sHR with IPTWs (95% CI)^a^	0.78 (0.70–0.86)	1.00 (reference)
Chronic kidney disease		
Total *n*	9620	9962
First hospital admission with a principal diagnosis of CKD, *n* (%)	87 (0.4)	157 (0.8)
Competing event, all‐cause death, *n* (%)	102 (0.5)	278 (1.4)
Follow‐up time in years, median (IQR)	1.4 (0.7–2.2)	1.7 (0.8–2.6)
Incidence rate, number of events per 1000 person‐years	6.0	8.9
Unadjusted sHR (95% CI)	0.69 (0.53–0.90)	1.00 (reference)
Adjusted sHR (95% CI)^a^	0.81 (0.62–1.07)	1.00 (reference)
Adjusted sHR with IPTWs (95% CI)^a^	0.83 (0.73–0.94)	1.00 (reference)

Abbreviations: ARF, acute renal failure; CI, confidence interval; CKD, chronic kidney disease; DPP‐4i, dipeptidyl peptidase‐4 inhibitor; IQR, interquartile range; IPTW, inverse probability of treatment weights; sHR, subdistribution hazard ratio; SGLT‐2i, sodium glucose cotransporter‐2 inhibitor.

^a^ Excluded people with missing data from the Socio‐Economic Index for Areas: Index of Relative Socioeconomic Disadvantage.

A lower proportion of the SGLT‐2i initiators (0.5%) died from any cause compared to the DPP‐4i initiators (1.3%) in the cohort where ARF was reported. A similar trend was seen in the cohort where CKD was reported with 0.5% of the SGLT‐2i initiators dying compared to 1.4% of DPP‐4i initiators (Table 2).

### 3.2 Acute renal failure and chronic kidney disease

The incidence rate of first hospitalization for ARF during the follow‐up period was 12.3 events per 1000 person‐years among SGLT‐2i initiators and 18.9 events per 1000 person‐years among DPP‐4i initiators. In the adjusted model, SGLT‐2i initiators had a 22% lower rate of first hospitalization for ARF compared with DPP‐4i initiators (adjusted sHR with IPTW 0.78; 95% CI 0.70–0.86) (Table 2). The incidence rate of first hospitalization for CKD was 6.0 events per 1000 person‐years among SGLT‐2i initiators and 8.9 events per 1000 person‐years among DPP‐4i initiators. In the adjusted model, SGLT‐2i initiators had a 17% lower rate of first hospitalization for CKD compared with DPP‐4i initiators (adjusted sHR with IPTW 0.83; 95% CI 0.73–0.94) (Table 2).

## 4 DISCUSSION

We demonstrated that initiation of SGLT‐2is versus DPP‐4is at hospital discharge was associated with a 22% reduction in the rate of readmission for ARF and 17% reduction in rate of CKD admissions in people who initiated SGLT‐2is versus DPP‐4is. These findings from real‐world data enhance what we know from randomized controlled trials (RCTs)^1^, ^2^, ^3^, ^7^, ^8^ and other observational studies^5^, ^18^ and suggest that SGLT2i do not cause ARF or have acute detriments on kidney function in people without severely reduced eGFR.

This was the first Australian study to demonstrate the real‐world benefits of SGLT‐2is versus DPP‐4is for reducing ARF and CKD. There are numerous reasons that explain how our real‐world data enhances what we know from RCTs. First, we were able to adjust for a range of clinically important covariates to minimize confounding by comorbidity and channeling bias by excluding people with prior medication use and comorbidities that predispose to ARF or CKD.^30^ Many prior studies did not exclude this prior medication use and comorbidities. Second, the study analyzed data for a large state‐wide cohort over a 6‐year period (July 2012 to June 2018). All patients who were hospitalized were included (there was no opt‐out option), which gave an accurate representation of the Victorian population.

Our study results are in alignment with previous RCTs and several other observational studies. An observational study in Canada including 39 094 people compared the 90 day risk of ARF in new initiators of SGLT‐2is and DPP‐4is, finding a 21% reduced risk of ARF for the SGLT‐2i group compared to the DPP‐4i group (risk ratio 0.79 (95% CI 0.64–0.98).^13^ Similarly, Cahn et al reported a 53% (95% CI 20–73) reduced risk of ARF in SGLT‐2i versus DPP‐4i users in their overall population.^5^ Some previous studies have reported no significant effect of SGLT‐2is on ARF and have used any oral glucose lowering drug,^19^ GLP1 agonist,^20^ DPP‐4i/GLP1 agonist/gliclazide,^4^ or no use as a comparator group.^22^ Different comparators may explain some of the differences between our results and the findings from these studies. Previous RCTs have shown that SGLT‐2is are associated with reduced rates of progression of CKD incidence compared to DPP‐4is.^9^, ^10^, ^12^, ^13^, ^14^, ^29^

To summarize major trials in this space, the Canagliflozin and Renal Events in Diabetes with Established Nephropathy Clinical Evaluation (CREDENCE) trial compared people with type 2 diabetes and CKD to receive canagliflozin or placebo. The incidence of end‐stage kidney disease was lowered by 34% in the canagliflozin group compared to the placebo group (HR 0.66 (95% CI 0.54–0.86).^31^ In an RCT by Pasternak et al^13^ and by Pollock et al,^9^ the annual decline in eGFR slowed significantly after SGLT‐2i therapy was initiated.^9^, ^13^ Similarly, DAPA‐CKD^10^ showed among patients with CKD, the risk of a combined end point of at least 50% sustained decline in eGFR, end‐stage kidney disease, or death from renal causes being 0.56 (95% CI, 0.45–0.68).^10^

Our study had several limitations. First, the cohort included only people discharged from hospital, which limits generalizability. People who are hospitalized are usually sicker and have more comorbidities than the general population of people with type 2 diabetes. If the outcomes (AKI or CKD) were identified in the community they would have been missed as we are using only hospitalization data. However, this should be consistent across both groups and not specific to one medication class. Second, we did not have access to data on type 2 diabetes duration. However, we used DCSI as a surrogate for type 2 diabetes severity, which usually increases with duration. Third, during the study period, SGLT‐2is were contraindicated in people with impaired renal function (an eGFR <45 mL/min/1.73 m^2^). As the study had no access to eGFR, to address this we excluded people who had taken linagliptin, the preferred DPP‐4i for use in renal impairment. It is also possible that there have been evolutions in SGLT‐2i prescribing practices since 2018 and that clinicians may be more likely to prescribe SGLT‐2i in patients at risk of renal failure now than in 2013–2018. Fourth, it is unclear if people took the medications as directed. Adherence to the use of SGLT‐2 inhibitors and DPP‐4 inhibitors was not measured; however, we are looking at protective effects, hence using an intention to treat analysis (not following up for adherence) is conservative. Fifth, the study was unable to ascertain whether people were dispensed SGLT‐2is or DPP‐4is outside of the PBS reimbursement system. However, non‐PBS use is likely to have been rare due to higher costs to the patient. Finally, initiation of SGLT‐2i or DPP‐4is while in hospital for the index admission was not captured, and the date of first use could have been before the date of discharge. This could be a source of bias, in that people initiated while in hospital who have an acute kidney event while still in hospital, might have that medication withdrawn. Thus, the population of those who are discharged on the medication are enriched for people not at risk of an early acute kidney event.

The lack of specific clinical data, such as baseline renal function being not available, is a major limitation in analyzing the risk of kidney outcomes associated with medication usage. However, this is common for most studies using administrative data. Administrative data can still provide useful results without specific laboratory results. Perhaps future studies using electronic hospital records could address this.

There is a potential of under‐reporting of comorbidities in our data, because hospital admission data captures only severe forms of the disease, or that these patients have different treatment approaches depending on their comorbidities. Using our two‐pronged approach (both hospital coded diagnoses and medication dispensing) would be expected to be superior to using either one by itself.

One discrepancy is that heart failure prevalence is the same between groups; however, the proportions given heart failure drugs (such as aldosterone antagonist and digoxin) are different between groups. This could be due to the proportion that have heart failure with reduced ejection fraction compared to those with heart failure with preserved ejection fraction, where the treatment options are dramatically different. A limitation of the dataset is the specific type of heart failure is unavailable.

### 4.1 Implications for clinicians

This study found that in adults >30 years of age with type 2 diabetes, SGLT‐2is, compared to DPP‐4is, are associated with a reduced risk of being rehospitalized with ARF and CKD. This study contributes information toward understanding SGLT‐2i renal outcomes and this result adds to the mounting evidence that is likely to assuage clinicians' concerns that SGLT‐2i initiation is linked to ARF.

## AUTHOR CONTRIBUTIONS

Kate E. D. Ziser, Stephen Wood, George S. Q. Tan, Jedidiah I. Morton, Jonathan E. Shaw, J. Simon Bell, and Jenni Ilomaki made substantial contributions to study conception and design, acquisition of data or analysis and interpretation of the data. J. Simon Bell and Jenni Ilomaki obtained grant funding. All authors drafted (Kate E. D. Ziser) or revised (Stephen Wood, George S. Q. Tan, Jedidiah I. Morton, Jonathan E. Shaw, J. Simon Bell, and Jenni Ilomaki) the article for important intellectual content. Kate E. D. Ziser, Stephen Wood, George S. Q. Tan, Jedidiah I. Morton, Jonathan E. Shaw, J. Simon Bell, and Jenni Ilomaki approved the final version to be published.

## FUNDING INFORMATION

The authors gratefully acknowledge funding provided by the Dementia Australia Research Foundation – Yulgilbar Innovation Grant.

## DISCLOSURE

J. Simon Bell has received grant funding or consulting funds from the National Health and Medical Research Council, Medical Research Future Fund, Victorian Government Department of Health and Human Services, Dementia Australia Research Foundation, Yulgilbar Foundation, Aged Care Quality and Safety Commission, Dementia Centre for Research Collaboration, Pharmaceutical Society of Australia, Society of Hospital Pharmacists of Australia, GlaxoSmithKline Supported Studies Programme, Amgen, and several aged care provider organizations unrelated to this work. All grants and consulting funds were paid to the employing institution. Jonathan E. Shaw has received honoraria for lectures and for advisory boards from: Astra Zeneca; Sanofi; Novo Nordisk; MSD; Eli Lilly; Pfizer; Roche; Mylan; Boehringer Ingelheim; Zuellig; Roche. Kate E. D. Ziser, George S. Q. Tan, Jedidiah I. Morton, and Stephen Wood have no relationships or activities to disclose. Jenni Ilomaki has received funding or consulting funds from the National Health and Medical Research Council, Medical Research Future Fund, Victorian Government Department of Health and Human Services, Dementia Australia Research Foundation, Yulgilbar Foundation, National Breast Cancer Foundation, Amgen, and AstraZeneca. All grants and consulting funds were paid to the employing institution.

## Data Availability

The data that support the findings of this study are available from Australian Institute of Health and Welfare and the Centre for Victorian Data Linkage upon reasonable request.

## APPENDIX A

**TABLE A1:** Cause of index hospitalization in patients initiated SGLT‐2i or DPP‐4i.

Cause of index hospitalization	Frequency SGLT‐2i *n* (%)	Frequency DPP‐4i *n* (%)
Diabetes complications	392 (4.1)	320 (3.2)
Chest pain	441 (4.6)	302 (3.0)
Myocardial infarction	244 (2.5)	213 (2.1)
Cancer	299 (3.1)	419 (4.2)
Cataract	213 (2.2)	310 (3.1)
Angina	221 (2.3)	184 (1.8)
Obstructive sleep apnea syndrome	181 (1.9)	109 (1.3)
Atherosclerotic heart disease	155 (1.6)	128 (1.5)
Carpal tunnel syndrome	144 (1.5)	119 (1.4)
Screening for malignant neoplasm of intestine	143 (1.5)	132 (1.6)
Knee osteoarthritis	142 (1.5)	150 (1.8)
Gastrointestinal hemorrhage	130 (1.4)	109 (1.3)
Abdominal pain	130 (1.4)	143 (1.7)
GORD with esophagitis	107 (1.1)	84 (1.0)
Stroke	73 (0.8)	105 (1.1)
Others*	6605 (68.7)	7135 (71.6)
Total	9620	9962

Abbreviations: DPP‐4i, dipeptidyl peptidase‐4 inhibitor; SGLT‐2i, sodium glucose cotransporter‐2 inhibitor.

* Other causes of index hospitalization with frequency of <2% in study cohort.

**TABLE A2:** Anatomical therapeutic chemical (ATC) codes to identify medications.

Drug class	ATC code
Exposure medications	
SGLT‐2is	A10BK, A10BD07–08, A10BD15–16, A10BD19–21, A10BD23–25, A10BX11
DPP‐4is	A10BH (excluding A10BH05), A10BD10, A10BD12–13, A10BD18, A10BD21–22, A10BD24–25.
Baseline medications^a^	
ACEI/ARB	C09A, C09B, C09C, C09D (exclude C09DX04)
Beta‐blockers	C07
Calcium channel blockers	C08C
Lipid‐lowering medications	C10AA, C10BA, C10BX, C10AB, C10AC, C10AD, C10AX
Aldosterone antagonists	C03DA
Digoxin	C01AA05
Oral anticoagulant	B01AA
Antipsychotics	N05A
Antiplatelets	B01AC A10A
Anticholinergics	N06D

Abbreviations: ACE, angiotensin converting enzyme inhibitor; ARB, angiotensin 2 receptor blocker; DPP‐4i, dipeptidyl peptidase‐4 inhibitor; SGLT‐2i, sodium glucose cotransporter‐2 inhibitor.

^a^ Baseline medications are identified using a fixed 1‐year period prior to first dispensing date of exposure medication.

**TABLE A3:** Specification of relevant prior medical conditions.

Medical condition	ICD‐10‐AM	Time frame
Acidosis	E10.1, E11.1, E12.1, E13.1, E14.1, E87.2	2006 until first dispensing of exposure/comparator
Angina	I20.0, I20.1, I20.8, I20.9	2006 until first dispensing of exposure/comparator
Atrial fibrillation	I48	2006 until first dispensing of exposure/ comparator
Cancer	C00‐C99	Within 1 year prior to first dispensing of exposure/comparator (inclusive).
COPD and asthma	J44‐46	2006 until first dispensing of exposure/comparator
Diabetic eye complications	H28.0, H35.8, H36.0, E10.3, E11.3, E12.3, E13.3, E14.3	2006 until first dispensing of exposure/comparator
Diabetic foot/peripheral angiopathy	E11.6B, M14.2, M14.6, M90.8, L98.4, E10.5, E11.5, E12.5, E13.5, E14.5	2006 until first dispensing of exposure/comparator
Diabetic mono/polyneuropathy	G99.0, G59.0, G63.2, E10.4, E11.4, E12.4, E13.4, E14.4	2006 until first dispensing of exposure/comparator
Diabetes with several/unspecified complications	E11.6, E10.6, E13.6, E14.6, E10.7, E11.7, E12.7, E13.7, E14.7, E10.8, E11.8, E12.0, E12.8, E13.8, E14.8	2006 until first dispensing of exposure/comparator
Heart failure	I50	2006 until first dispensing of exposure/comparator
Hypertension	I10‐I15	2006 until first dispensing of exposure/comparator
Keto−/lactate acidosis	E10.1, E11.1, E12.1, E13.1, E14.1, E87.2	Within 1 year prior to first dispensing of exposure/comparator (inclusive).
Lower limb amputations	Z89	Within 1 year prior to first dispensing of exposure/comparator (inclusive).
Peripheral artery disease	I70‐I73	2006 until first dispensing of exposure/comparator
Severe hypoglycemia	E10.0, E11.0, E12.0, E13.0, E14.0, E11.6A, E16.0–2	Within 1 year prior to first dispensing of exposure/comparator (inclusive).
Stroke	I60‐I64	2006 until first dispensing of exposure/comparator

Abbreviations: COPD, chronic obstructive pulmonary disease; ICD‐10‐AM, *International Classification of Diseases, Tenth Revision, Australian Modification*.

## References

[1] McMurray JJV , DeMets DL , Inzucchi SE , et al. DAPA‐HF committees and investigators. A trial to evaluate the effect of the sodium glucose co‐transporter 2 inhibitor dapagliflozin on morbidity and mortality in patients with heart failure and reduced left ventricular ejection fraction (DAPA‐HF). Eur J Heart Fail. 2019;21:665‐675. doi:10.1002/ejhf.1432 30895697 PMC6607736

[2] Santos‐Gallego CG , Vargas‐Delgado AP , Requena‐Ibanez JA , et al. EMPA‐TROPISM (ATRU‐4) investigators. Randomized trial of empagliflozin in nondiabetic patients with heart failure and reduced ejection fraction. J Am Coll. 2021;77(3):243‐255. doi:10.1016/j.jacc.2020.11.008 33197559

[3] Zinman B , Wanner C , Lachin JM , Fitchett D , Bluhmki E , Hantel S . Empagliflozin, cardiovascular outcomes, and mortality in type 2 diabetes. N Engl J Med. 2015;373:2117‐2128. doi:10.1056/NEJMoa1504720 26378978

[4] Fadini GP , Solini A , Manca ML , et al. Effectiveness of dapagliflozin versus comparators on renal endpoints in the real world: a multicentre retrospective study. Diabetes Obes Metab. 2019;21(2):252‐260. doi:10.1111/dom.13508 30136354 PMC6585815

[5] Cahn A , Melzer‐Cohen C , Pollack R , Chodick G , Shalev V . Acute renal outcomes with sodium‐glucose co‐transporter‐2 inhibitors: real‐world data analysis. Diabetes Obes Metab. 2019;21(2):340‐348. doi:10.1111/dom.13532 30207040

[6] US Food and Drug Administration . FDA Approves Treatment for Chronic Kidney Disease. 2021 Available from: https://www.fda.gov/news‐events/press‐announcements/fda‐approves‐treatment‐chronic‐kidney‐disease. Accessed 26 May 2021

[7] Heerspink HJL , Karasik A , Thuresson M , et al. Kidney outcomes associated with use of SGLT2 inhibitors in real‐world clinical practice (CVD‐REAL 3): a multinational observational cohort study. Lancet Diabetes Endocrinol. 2020;8(1):27‐35. doi:10.1016/S2213-8587(19)30384-5 31862149

[8] Kaze AD . Association of SGLT2 inhibitors with cardiovascular, kidney, and safety outcomes among patients with diabetic kidney disease: a meta‐analysis. Cardiovasc Diabetol. 2022;21:47. doi:10.1186/s12933-022-01476-x 35321742 PMC9491404

[9] Pollock C , Stefánsson B , Reyner D , et al. Albuminuria‐lowering effect of dapagliflozin alone and in combination with saxagliptin and effect of dapagliflozin and saxagliptin on glycaemic control in patients with type 2 diabetes and chronic kidney disease (DELIGHT): a randomised, double‐blind, placebo‐controlled trial. Lancet Diabetes Endocrinol. 2019;7(6):429‐441. doi:10.1016/S2213-8587(19)30086-5 30992195

[10] Heerspink H , Cherney D , Postmus D , et al. A pre‐specified analysis of the dapagliflozin and prevention of adverse outcomes in chronic kidney disease (DAPA‐CKD) randomized controlled trial on the incidence of abrupt declines in kidney function. Kidney Int. 2022;101(1):174‐184. doi:10.1016/j.kint.2021.09.005 34560136

[11] Nagasu H , Yano Y , Kanegae H , et al. Kidney outcomes associated with SGLT2 inhibitors versus other glucose‐lowering drugs in real‐world clinical practice: the Japan chronic kidney disease database. Diabetes Care. 2021;44(11):2542‐2551. doi:10.2337/dc21-1081 34593566 PMC8546274

[12] Sugiyama S , Jinnouchi H , Yoshida A , et al. Renoprotective effects of additional SGLT2 inhibitor therapy in patients with type 2 diabetes mellitus and chronic kidney disease stages 3b‐4: a real world report from a Japanese specialized diabetes care center. J Clin Med Res. 2019;11(4):267‐274. doi:10.14740/jocmr3761 30937117 PMC6436561

[13] Iskander C , Cherney DZ , Clemens KK , et al. Use of sodium–glucose cotransporter‐2 inhibitors and risk of acute kidney injury in older adults with diabetes: a population‐based cohort study. CMAJ. 2020;192(14):E351‐E360. doi:10.1503/cmaj.191283 32392523 PMC7145366

[14] Rampersad C , Kraut E , Whitlock RH , et al. Acute kidney injury events in patients with type 2 diabetes using SGLT2 inhibitors versus other glucose‐lowering drugs: a retrospective cohort study. Am J Kidney Dis. 2020;76(4):471‐479.e1. doi:10.1053/j.ajkd.2020.03.019 32464161

[15] Ueda P , Svanström H , Melbye M , et al. Sodium glucose cotransporter 2 inhibitors and risk of serious adverse events: nationwide register based cohort study. BMJ. 2018;363:k4365. doi:10.1136/bmj.k4365 30429124 PMC6233755

[16] Pasternak B , Wintzell V , Melbye M , et al. Use of sodium‐glucose co‐transporter 2 inhibitors and risk of serious renal events: Scandinavian cohort study. BMJ. 2020;369:m1186. doi:10.1136/bmj.m1186 32349963 PMC7188014

[17] Zhuo M , Paik JM , Wexler DJ , Bonventre JV , Kim SC , Patorno E . SGLT2 inhibitors and the risk of acute kidney injury in older adults with type 2 diabetes. Am J Kidney Dis. 2022;79(6):858‐867. doi:10.1053/j.ajkd.2021.09.015 34762974 PMC9079190

[18] Schneeweiss S , Patorno E . Conducting real‐world evidence studies on the clinical outcomes of diabetes treatments. Endocr Rev. 2021;42(5):658‐690. doi:10.1210/endrev/bnab007 33710268 PMC8476933

[19] Kennedy‐Martin T , Curtis S , Faries D , Robinson S , Johnston J . A literature review on the representativeness of randomized controlled trial samples and implications for the external validity of trial results. Trials. 2015;3(16):495. doi:10.1186/s13063-015-1023-4 PMC4632358 26530985

[20] Sen A , Goldstein A , Chakrabarti S , et al. The representativeness of eligible patients in type 2 diabetes trials: a case study using GIST 2.0. J Am Med Inform Assoc. 2018;25:239‐247. doi:10.1093/jamia/ocx091 29025047 PMC7378875

[21] Mabbott V , Storey P . Australian Statistics on Medicines 2015. 2016 Available from: https://www.pbs.gov.au/info/statistics/asm/asm-2015

[22] WHO Collaborating Centre for Drug Statistics Methodology . Norweign Institute of Public Health. ATC/DDD Index; 2022 Available from: https://www.whocc.no/atc_ddd_index/

[23] Australian Type 2 Diabetes Management Algorithm . Australian Diabetes Society. 2020 Available from: http://t2d.diabetessociety.com.au/documents/qRkc5Qkv.pdf

[24] Dugan J , Shubrook J . International classification of diseases, 10th revision, coding for diabetes. Clin Diabetes. 2017;35(4):232‐238. 29109613 10.2337/cd16-0052 PMC5669129

[25] Australian Bureau of Statistics, Socio‐Economic Index for Areas (SEIFA) . Technical Paper. Canberra; 2016 Available from: https://www.abs.gov.au/

[26] Morton JI , Ilomӓki J , Magliano DJ , Shaw JE . The association of socioeconomic disadvantage and remoteness with receipt of type 2 diabetes medications in Australia: a nationwide registry study. Diabetologia. 2021;64:349‐360. doi:10.1007/s00125-020-05304-3 33078206

[27] Sriphrapradang C , Thewjitcharoen Y , Buranapin S , et al. Effectiveness and safety of sodium–glucose co‐transporter‐2 inhibitors in Thai adults with type 2 diabetes mellitus: a real‐world study. Curr Med Res Opin. 2020;36(10):1601‐1610. doi:10.1080/03007995.2020.1808454 32776785

[28] Zhou FL , Watada H , Tajima Y , et al. Identification of subgroups of patients with type 2 diabetes with differences in renal function preservation, comparing patients receiving sodium‐glucose co‐transporter‐2 inhibitors with those receiving dipeptidyl peptidase‐4 inhibitors, using a supervised machine‐learning algorithm (PROFILE study): a retrospective analysis of a Japanese commercial medical database. Diabetes Obes Metab. 2019;21(8):1925‐1934. doi:10.1111/dom.13753 31050099 PMC6771907

[29] Wood SJ , Bell JS , Magliano DJ , Shaw JE , Cesari M , Ilomaki J . Effectiveness of sodium‐glucose Cotransporter‐2 inhibitors vs. dipeptidyl Peptidase‐4 inhibitors in frail people with diabetes who were recently hospitalized. Front Pharmacol. 2022;12(13):886834. doi:10.3389/fphar.2022.886834 PMC9315378 35903329

[30] Brookhart MA , Wyss R , Layton JB , Stürmer T . Propensity score methods for confounding control in nonexperimental research. Circ Cardiovasc Qual Outcomes. 2103;6(5):604‐611. doi:10.1161/CIRCOUTCOMES.113.000359 PMC4032088 24021692

[31] Perkovic V , Jardine MJ , Neal B , et al. Canagliflozin and renal outcomes in type 2 diabetes and nephropathy. N Engl J Med. 2019;380:2295‐2306. doi:10.1056/NEJMoa1811744 30990260

